# ELF3, ELF5, EHF and SPDEF Transcription Factors in Tissue Homeostasis and Cancer

**DOI:** 10.3390/molecules23092191

**Published:** 2018-08-30

**Authors:** Ian Y. Luk, Camilla M. Reehorst, John M. Mariadason

**Affiliations:** 1 Olivia Newton-John Cancer Research Institute, Heidelberg, Victoria 3084, Australia; ian.luk@onjcri.org.au (I.Y.L.); camilla.reehorst@onjcri.org.au (C.M.R.); 2 School of Cancer Medicine, La Trobe University, Bundoora, Victoria 3086, Australia

**Keywords:** ELF3, ELF5, EHF, SPDEF, transcription factor, cancer, J0101

## Abstract

The epithelium-specific ETS (ESE) transcription factors (ELF3, ELF5, EHF and SPDEF) are defined by their highly conserved ETS DNA binding domain and predominant epithelial-specific expression profile. ESE transcription factors maintain normal cell homeostasis and differentiation of a number of epithelial tissues, and their genetic alteration and deregulated expression has been linked to the progression of several epithelial cancers. Herein we review the normal function of the ESE transcription factors, the mechanisms by which they are dysregulated in cancers, and the current evidence for their role in cancer progression. Finally, we discuss potential therapeutic strategies for targeting or reactivating these factors as a novel means of cancer treatment.

## 1. Introduction

The epithelium-specific ETS (ESE) transcription factors are a sub-group of the larger E26 transformation-specific (ETS) transcription factor family, of which there are 28 members in humans and 27 in mice [1]. All ETS factors share a conserved 85 amino acid (aa) ETS DNA binding domain [2], and regulate gene expression by binding to the core *GGAA/T* regulatory sequence where the H3 helix of the ETS domain inserts into the major groove of DNA [2]. 

**Figure 1:**
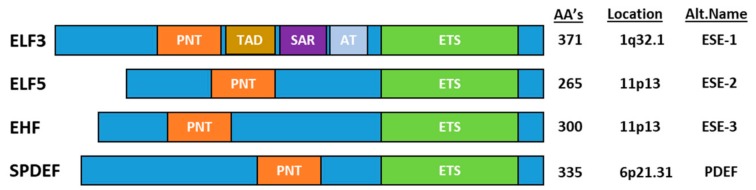
Schematic representation of the epithelial-epecific ETS transcription factors. Adapted from Feldman et al., 2003 [5] and Archer et al., 2017 [6].

The ESE transcription factors can be divided into two groups based on the homology of the ETS DNA binding domain–(1) ELF3, ELF5 and EHF and (2) SPDEF–but are grouped together based on their common epithelial-specific expression profile. In addition to the ETS domain, all ESE members share an N-terminal Pointed (PNT) domain comprising ~80 aa’s [3], which is similar to the evolutionarily conserved sterile alpha motif (SAM) domain (Figure 1). SAM/(PNT) domains perform a range of functions, including mediating protein-protein interactions, kinase docking, RNA-binding and lipid molecule interactions, as well as transcriptional activation [4]. 

ESE transcription factors play a key role in normal development, as well as differentiation and homeostasis of specific epithelial tissues. We will review these processes by describing the expression profile of ESE factors in normal tissues, focussing on the phenotypes of ESE factor knockout mice which have been described in detail for *Elf3*, *Elf5* and *Spdef*. ESE factors have also been implicated in the pathogenesis of a wide range of cancers, with both oncogenic and tumour suppressive roles described depending on the specific ESE factor and tumour type. We also review the current knowledge regarding the role of each ESE factor in cancer progression, focussing on their genetic alteration, altered expression, association with outcome, and mechanisms of action.

### 1.1. Role of ELF3 in Normal Tissue Homeostasis 

**Figure 2:**
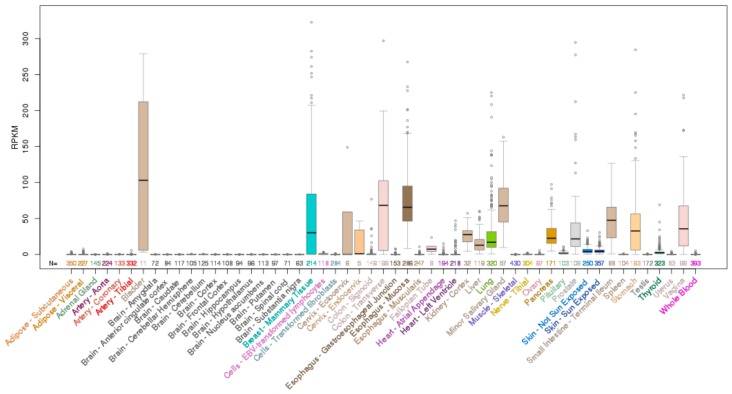
Expression of *ELF3* mRNA across normal human tissues. Data obtained from University of California Santa Cruz (UCSC) genome browser.

*ELF3* is located on chromosome 1q32.1 and encodes a 371 aa protein. Analysis of the mRNA expression profile of *ELF3* across normal human tissues reveals highest expression in the gastrointestinal tract (esophagus, large and small intestine and stomach), with strong expression also in the salivary gland, bladder, liver and prostate (Figure 2). As the role of ELF3 in normal epithelial tissues has been previously reviewed [7], we only briefly summarize these findings below. 

#### 1.1.1. *Elf3* Knockout Mice

Whole body knockout of *Elf3* results in embryonic lethality in ~30% of mice at ~E11.5 [8]. Of the *Elf3^−/−^* mice which are born, over half die prior to weaning due to development of diarrhea and a wasted phenotype suggestive of malnourishment, although interestingly, those animals which survive the first 6 weeks gain weight normally [8]. 

#### 1.1.2. ELF3 in the Intestinal Epithelium

A likely cause of the wasted phenotype of *Elf3^−/−^* mice is the abnormal differentiation of the small intestinal epithelium, where the enterocytes contain fewer microvilli and display loss of polarity, and the number of differentiated goblet cells is reduced. *TgfβRII* mRNA expression is reduced in the intestinal epithelium of *Elf3^−/−^* mice, and crossing *Elf3^−/−^* mice with intestinal-specific *TgfβRII* transgenic mice rescues the *Elf3^−/−^* phenotype, demonstrating that ELF3 maintains intestinal cell differentiation through activating TGFβ-signalling [9].

#### 1.1.3. ELF3 in the Lung Epithelium

*Elf3^−/−^* mice also show delayed bronchial epithelial regeneration following naphthalene-induced Clara cell injury, indicating a role for Elf3 in regulating proliferation and differentiation in this context [10]. As observed in the intestine, expression of TgfβRII, which is a key factor required for epithelial differentiation is reduced in the bronchial epithelium of *Elf3^−/−^* mice, which may contribute to the phenotype [10]. 

#### 1.1.4. ELF3 in the Urothelium

ELF3 protein is expressed in differentiated superficial urothelial cells and ELF3 mRNA and protein expression is increased during PPARγ-induced differentiation of normal human urothelial cells in vitro [11]. ELF3 knockdown in this model attenuates expression of key urothelial differentiation-inducing transcription factors (FOXA1 and GRHL3), and markers of urothelial differentiation, establishing a direct requirement for ELF3 for urothelial differentiation [11]. 

#### 1.1.5. ELF3 in Squamous Epithelium

*ELF3* mRNA is induced during terminal differentiation of the epidermis [12], and directly drives expression of the terminal differentiation marker SPRR2A through binding to consensus ETS binding sites within its promoter [12]. ELF3 also induces expression of SPRR2A in oesophageal and cervical squamous epithelial cell lines [13], while in parallel repressing expression of the early marker of differentiation, KRT4, indicative of a dual function in promoting squamous epithelial differentiation. 

*Elf3* mRNA is also expressed in the differentiated mouse corneal epithelium and is induced during human corneal epithelial differentiation in vitro. ELF3 overexpression transactivates the *KRT12* promoter, and ELF3 knockdown reduces morphological features of squamous epithelial differentiation, establishing a direct role in the differentiation process [14].

#### 1.1.6. ELF3 in Non-Epithelial Cells

While ELF3 is primarily expressed in epithelial cells, it also plays a role in vascular inflammation and remodeling [15]. ELF3 mRNA and protein is induced in primary endothelial cells and vascular smooth muscle cells upon stimulation with proinflammatory cytokines or angiotensin II, and *Elf3^−/−^* mice stimulated with angiotensin II display increased T cell and macrophage infiltration into the vessel wall, increased vascular thickening and fibrosis, and have an exaggerated systolic blood pressure response. Induction of the Elf3 target gene, nitric oxide synthase 2 (*Nos2*), is also attenuated in vascular smooth muscle cells in *Elf3^−/−^* mice, raising the possibility that Elf3 may normally act to balance inflammatory responses in this tissue by inducing nitric oxide production by driving *Nos2* expression [15]. 

Recently, a role for ELF3 in contributing to cartilage degradation was demonstrated in a model of post-traumatic osteoarthritis. ELF3 protein is overexpressed in the cartilage of patients with osteoarthritis, and drives expression of the IL-1β-induced genes *MMP13*, *NOS2*, and *PTGS2/COX2* in chondrocytes [16]. Conditional deletion of *Elf3* in mouse chondrocytes results in decreased IL-1β-mediated induction of *Mmp13* and *Nos2*, and these mice undergo less cartilage loss in a model of osteoarthritis. Conversely, chondrocyte-specific *Elf3* overexpression induces the opposite effect [17].

Finally, Elf3 is suggested to regulate allergic airway inflammation, as *Elf3^−/−^* mice display an impaired Th17 response to pulmonary inflammatory challenge. Specifically, *Elf3^−/−^* dendritic cells produce lower levels of IL-12, a key cytokine required for Th1 differentiation, resulting in an exaggerated Th2 inflammatory response [18]. 

### 1.2. Role of ELF3 in Cancer 

**Figure 3:**
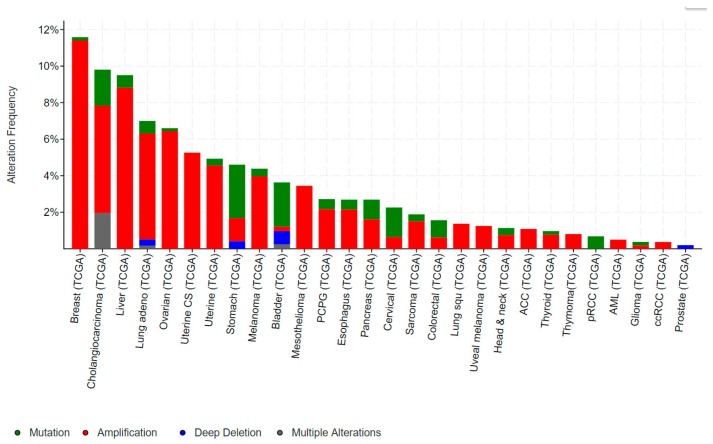
Frequency of genomic alterations in *ELF3* in human cancers. Data obtained from cancer bioportal [19,20]. Only showing cancers in which >50 cases were analysed.

Mutations in *ELF3*, particularly inactivating mutations, occur in bladder, cervical, ovarian and a number of gastrointestinal cancers, including cancers of the biliary tract, stomach and colon. Conversely, gene amplification of *ELF3* occurs in some of these same cancers (colorectal, gastric), as well as in breast and liver cancer (Figure 3). 

#### 1.2.1. ELF3 in Bladder Cancer

A recent exome-sequencing study of bladder cancer performed by The Cancer Genome Atlas (TCGA) consortium reported mutations in *ELF3* in ~6% of both superficial and invasive tumours, over half of which were inactivating mutations [21]. Comparatively, categorization of bladder cancers into luminal or basal subtypes identified more frequent *ELF3* mutations in luminal tumours [22,23]. While the inactivating mutations in *ELF3* would suggest a tumour suppressive role, the functional significance of these mutations in disease pathogenesis has yet to be directly established in cell line or animal models. 

#### 1.2.2. ELF3 in Ovarian Cancer (OC)

Similar to bladder cancer, inactivating and splice site mutations in *ELF3* occur in ~6% of mucinous OCs [24]. Furthermore, loss of ELF3 mRNA and protein expression is associated with poorer outcome in OC, collectively suggesting a tumour suppressive role [25]. Consistent with such a role, re-expression of ELF3 in OC cell lines with low endogenous expression inhibits proliferation in vitro and in vivo, and promotes mesenchymal to epithelial transition (MET). Conversely, ELF3 knockdown in OC cell lines induced epithelial to mesenchymal transition (EMT), suggesting ELF3 inhibits ovarian tumour progression by maintaining the epithelial state [25]. 

#### 1.2.3. ELF3 in Biliary Tract Cancers

Inactivating mutations in *ELF3* occur in ~6% of cholangiocarcinomas [26,27] and gallbladder cancers [28,29], and in ~10% of ampullary cancers [30,31], which arise from the Ampulla of Vater, the nipple-like projection into the duodenum into which the pancreatic and bile ducts open. Ampullary cancers originate from the different epithelial cell types present at the site, and *ELF3* mutations occur in both intestinal-type and pancreatobiliary-type ampullary adenocarcinomas. Furthermore, *ELF3* mutations in ampullary tumours occur in high allele frequencies suggesting they are likely founder mutations. The majority of *ELF3* truncating mutations in biliary tract cancer are heterozygous, suggesting ELF3 may act as a haplo-insufficient tumour suppressor [30]. Consistent with such a role, knockdown of ELF3 in an immortalized epithelial cell line of common bile duct origin increased cell invasion and motility, and promoted EMT [30], while re-expression of ELF3 in a cholangiocarcinoma cell line harbouring a heterozygous frameshift mutation inhibited proliferation [26].

#### 1.2.4. ELF3 in Gastric and Colorectal Cancer

*ELF3* is mutated in 2–4% of gastric cancers (GCs) and colorectal cancers (CRCs), and deleted in a further ~1% of GCs (Figure 3). As for bladder and biliary tract cancers, the majority of mutations are truncating, suggestive of a tumour suppressive role. Notably, the TCGA also identified *ELF3* amplifications in ~1–2% of GCs and CRCs, while a study by Wang et al. suggested this rate may be as high as 17% [32]. Wang et al. also reported increased ELF3 protein expression in CRC compared to the adjacent normal epithelium, and an association of high ELF3 protein and mRNA expression with worse overall survival. Overexpression of ELF3 in CRC cells increased cell proliferation and transcription of β-catenin, suggesting a novel means of driving WNT signaling in these tumours [32]. On the other hand, Yang et al. demonstrated that ELF3 co-localizes and physically interact with β-catenin in CRC cells [33]. Intriguingly, this complex forms in the cytoplasm, raising the possibility that ELF3 may act to sequester β-catenin away from the nucleus and dampen β-catenin-driven transcription. 

Additional studies, including experiments in animal models, are still required to resolve these discrepancies and definitively establish the role of ELF3 in gastric and CRC progression. 

#### 1.2.5. ELF3 in Cervical Cancer 

Frameshift mutations in *ELF3* occur in ~13% of cervical adenocarcinomas [34], which surprisingly express higher levels of *ELF3* mRNA compared to wild-type tumours. This may be due to futile overexpression of the mutant allele, or compensatory upregulation of the wild-type allele. Whether these mutations are homo or heterozygous, and the effect of these mutations on tumour initiation and progression, remains to be determined [34]. 

#### 1.2.6. ELF3 in Breast Cancer

Studies in breast cancer suggest ELF3 may function as either a tumour promoter or suppressor, depending on the molecular subtype of the disease. In Estrogen Receptor α (ERα) positive breast cancer, ELF3 directly binds and represses the transcriptional activity of ERα, suggestive of a tumour suppressive role. Ectopic expression of ELF3 also reduced ERα target gene expression, and decreased oestrogen-dependent proliferation of MCF7 cells, whereas ELF3 knockdown in the same cell line induced the opposite effects [35]. *ELF3* was also itself found to be an ERα target gene, suggesting it may act as a negative feedback regulator of ERα signaling [35]. Similarly, in triple negative breast cancer (TNBC), ELF3 mRNA expression is lower in primary tumours compared to normal breast epithelium, and further reduced in distant metastases [36]. Interestingly, sorting of TNBC organoids into more differentiated or stem-like populations revealed higher *ELF3* mRNA expression in the differentiated population. *ELF3* mRNA was also induced following pharmacological induction of TNBC cell differentiation, and knockdown and overexpression experiments demonstrated ELF3 was required for the differentiation-inducing effect of this drug treatment [36].

Conversely, ELF3 may have a pro-tumorigenic role in HER2^+^ tumours, where ELF3 is upregulated due to HER2-signaling driven activation of the ELF3 promoter [37,38]. HER2^+^ breast cancers with high *ELF3* mRNA expression have a worse outcome, and ELF3 knockdown in HER2^+^ breast cancer cell lines attenuated tumour growth through inhibition of AKT signaling [39]. ELF3 knockdown also inhibited the growth in HER2^+^ trastuzumab-resistant breast cancer cell lines [40].

#### 1.2.7. ELF3 in Prostate Cancer

ELF3 mRNA and protein expression are increased in primary prostate cancers compared to normal prostate tissue, and further increased in metastases [41]. *ELF3* is also amplified in 2–6% of tumours collectively suggesting a role in tumour progression. The high ELF3 expression in prostate cancers is driven, at least in part, by the pro-inflammatory cytokine IL-1β, in a NF-κB-dependent manner. In turn, ELF3 interacts with NF-κB and enhances NF-κB-driven transcription, creating a positive feedback loop which constitutively activates NF-κB and promotes prostate cancer progression. Notably, the subset of prostate cancers which express high levels of both ELF3 and nuclear NF-κB (p65) have a significantly worse prognosis [41]. Further to this, stable expression of ELF3 in LNCaP and 22RV1 prostate cancer cells increased proliferation, colony formation, cell migration and resistance to anoikis in vitro, and promoted formation of lung metastases in vivo [41].

In contrast, Shatnawi et al. reported a putative tumour suppressive function for ELF3 in prostate cancer [42]. While they did identify a subset of prostate cancers with increased *ELF3* mRNA expression, they observed downregulation of *ELF3* mRNA in the majority of primary tumours. Furthermore, a direct interaction between ELF3 and the androgen receptor (AR) was identified in LNCaP cells, which was enhanced upon treatment with an androgen agonist. Further studies established ELF3 as a repressor of AR activity contributing to the repression of AR target genes. ELF3 overexpression also attenuated the proliferation of AR agonist stimulated LNCaP cells in vitro, and repressed the growth of LNCaP xenografts in vivo [42]. Collectively, these findings suggest that loss of ELF3 represents an additional means of enhancing AR activity in prostate cancer cells. The basis for the disparity of these findings with those of Longoni et al. [41] is unclear, as the same cell line (LNCaP) was used in both studies, although notably, several of the effects reported by Shatnawi et al. were in the context of androgen agonist treatment. 

#### 1.2.8. ELF3 in Lung Cancer 

ELF3 mRNA and protein expression is elevated in non-small cell lung cancer (NSCLC) compared to corresponding normal lung tissue, with high expression associated with poor patient outcome. Furthermore, functional studies demonstrated that ELF3 knockdown inhibited proliferation, metastasis and EMT of lung cancer cell lines, and inhibited PI3K/AKT and ERK signalling [43], suggesting a tumour promoting role in these cancers. 

#### 1.2.9. ELF3 in Hepatocellular Cancer (HCC)

High protein expression of ELF3 in HCC is also associated with a poor patient outcome, and knockdown of ELF3 in HCC cell lines decreased proliferation, migration and invasion. ELF3 overexpression also promoted EMT as evidenced by the increased expression of mesenchymal markers N-Cadherin and fibronectin, and the EMT driver ZEB1. These effects were driven through ELF3-mediated suppression of miR-141-3p, which in turn resulted in overexpression of ZEB1 [44].

### 1.3. Role of ELF5 in Normal Tissue Homeostasis 

**Figure 4:**
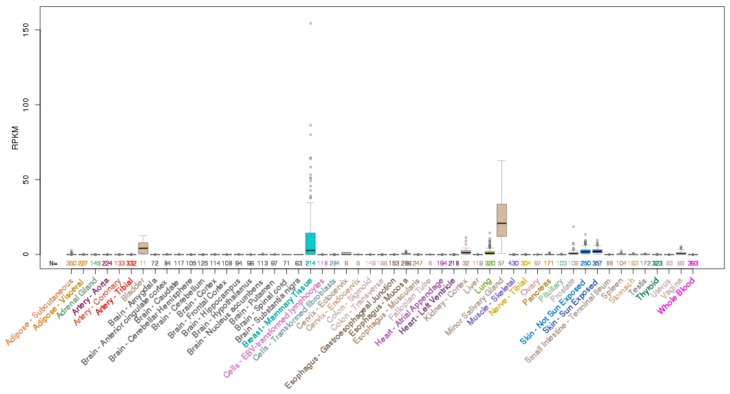
Expression of *ELF5* mRNA across normal human tissues. Data obtained from UCSC genome browser.

*ELF5* is located on chromosome 11p13 and encodes a 265 aa protein. Analysis of normal human tissues indicates *ELF5* mRNA expression is restricted to only a few epithelial tissues with highest expression in the salivary gland, breast and bladder, and some expression in the lung, kidney and skin (Figure 4). 

#### 1.3.1. *Elf5* Knockout Mice

Two groups have generated *Elf5* knockout mice and both demonstrated that constitutive *Elf5* deletion results in embryonic lethality [45,46]. *Elf5^−/−^* embryos implant at expected Mendelian ratios but no *Elf5^−/−^* embryos survive beyond E7.5. Subsequent studies demonstrated that *Elf5*-deficient embryos fail to develop the extraembryonic ectoderm due to terminal differentiation of trophoblast stem cells [46,47], resulting in severe patterning defects and failure of the embryos to undergo gastrulation [46]. 

#### 1.3.2. ELF5 in the Mammary Epithelium

Elf5 is required for the proliferation and differentiation of mammary alveolar epithelial cells during pregnancy and lactation, as loss of even one allele of *Elf5* (*Elf5^+/−^* mice) results in complete developmental arrest of the mammary gland [45]. These findings were confirmed in mammary-specific *Elf5^−/−^* mice where mammary epithelial cells had disorganized cell structures and reduced milk production [48]. Subsequent studies demonstrated that *Elf5* functions downstream of the prolactin receptor signaling pathway but upstream of STAT5 [48], both of which are required for differentiation of the mammary alveolar epithelium [49].

*Elf5* also plays a role in determining cell fate and stem cell function in the mammary epithelium, as mammary-specific *Elf5* deletion leads to an accumulation of mammary stem cells and the proportion of cells with dual luminal/basal properties [50]. *Elf5* deletion also resulted in EMT in the mammary epithelium at lactation day 1, indicating its requirement for maintenance of the epithelial state [51]. 

#### 1.3.3. ELF5 in the Kidney 

In the kidney, ELF5 is specifically expressed in the principal cell lineage of the collecting ducts and is induced by Notch signaling, a key regulator of this lineage. However, conditional deletion of *Elf5* in the developing collecting ducts had a minimal effect on principal cell differentiation, which may be due to compensatory activation of other transcription factors, including other ESE members such as *Ehf*, which also regulates aspects of principal cell differentiation [52]. 

#### 1.3.4. ELF5 in the Skin 

Analysis of *Elf5*-LacZ mice in which the *Elf5* coding region is replaced by the beta-galactosidase (LacZ) reporter revealed that Elf5 is expressed in the differentiated cells of the inner root sheath of the hair follicle [53]. *Elf5* is also upregulated during keratinocyte differentiation in vitro [54], however whether it is directly required for keratinocyte differentiation remains to be determined.

### 1.4. Role of ELF5 in Cancer 

**Figure 5:**
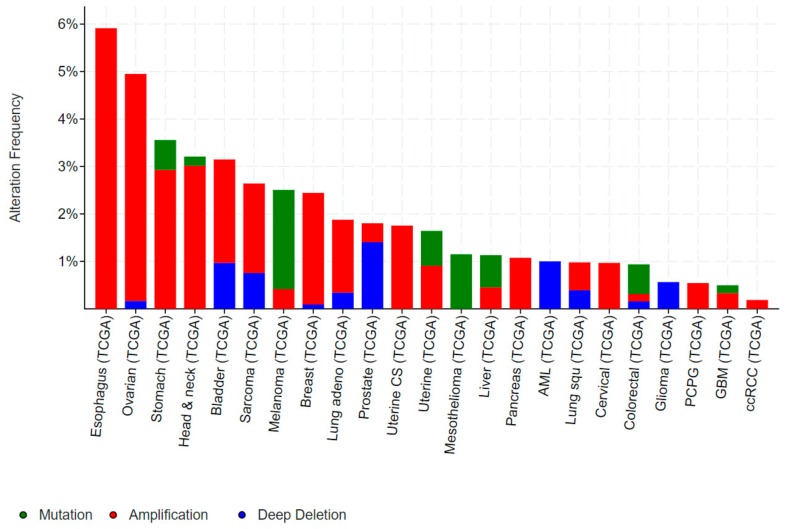
Genomic alterations in *ELF5* in human cancers. Data from cancer bioportal. Only showing cancers in which >50 cases were analysed.

Examination of publicly accessible genomic data reveals mutations in *ELF5* are relatively rare in human cancers. Missense mutations occur in melanoma, mesothelioma, uterine, stomach and colorectal cancers which are predominantly non-recurring missense of unknown function (Figure 5). 

**Figure 6:**
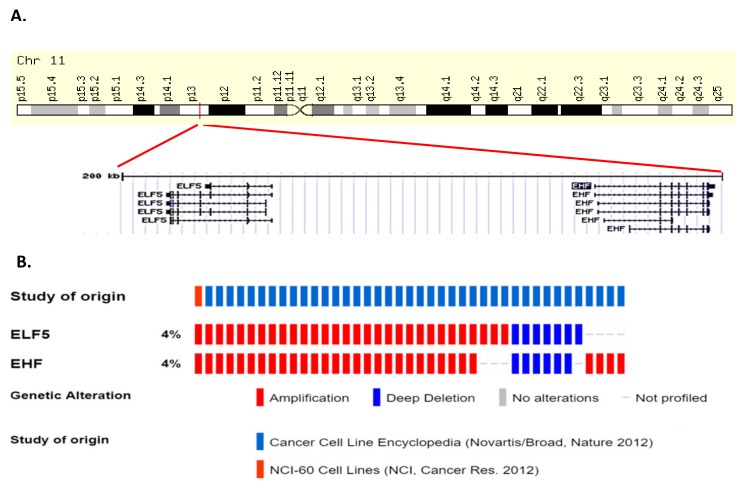
(**A**) Chromosomal location of *ELF5* and *EHF* at location 11p13. (**B**) Amplification and deletion pattern of *ELF5* and *EHF* across a panel of cancer cell lines.

Amplification of *ELF5* occur in cancers of the upper GI tract (oesophageal and stomach), ovary, head and neck, and breast in 2–6% of cases, while occasional deletions of *ELF5* occur in prostate, sarcoma, bladder, and lung cancers as well as acute myeloid leukemia (AML) and gliomas (Figure 5). *ELF5* is located within close proximity of the related ESE member *EHF* on chromosome 11p13 (Figure 6A), and analysis of a panel of various cancer cell lines indicates that *EHF* and *ELF5* are amplified or deleted in the same cases (Figure 6B), making identification of the driver gene in these cases challenging. 

#### 1.4.1. ELF5 in Breast Cancer

Both tumour promoting and suppressive roles for ELF5 have been reported in breast cancer, which may be linked to the molecular subtype of the disease. In TNBCs, high ELF5 protein expression is associated with worse outcome [55]. Comparatively, in the luminal A, luminal B, and HER2 subtypes, *ELF5* mRNA expression is reduced compared to the normal breast epithelium [51,56]. 

Direct evidence for a tumour suppressive role includes ELF5 silencing in epithelial-like T47D breast cancer cells inducing EMT, and ELF5 re-expression in mesenchymal-like MDA-MB-231 cells inducing epithelial features and reducing metastasis in vivo, at least in part by repressing expression of SNAIL2 [51]. Similarly, deleting *Elf5* in the MMTV-*neu* (*HER2*) background increased EMT markers and the formation of lung metastases [51], while transgenic overexpression of *Elf5* in the MMTV-*PyMT* model of luminal breast cancer inhibited cell proliferation [57]. 

ELF5 has also been suggested to regulate oestrogen sensitivity of breast cancers, as ELF5 repressed ER expression in MCF7 cells. Furthermore, *ELF5* mRNA expression is increased in MCF7 cells made resistant to tamoxifen by long term culture, and these cells were more dependent on ELF5 for their proliferation [58].

#### 1.4.2. ELF5 in Prostate, Urothelial, Ovarian and Renal Cancer

The majority of evidence generated to date indicates ELF5 plays a tumour suppressive role in prostate [59,60], bladder [61], ovarian [62] and renal cancer [63]. In prostate cancer, loss of ELF5 protein expression correlates with loss of expression of the epithelial marker E-Cadherin and increased expression of the mesenchymal marker N-Cadherin [59]. ELF5 knockdown in prostate cancer cell lines induced EMT, particularly in the presence of TGFβ, and ELF5 was subsequently shown to block EMT by inhibiting TGFβ signaling through binding and suppressing *SMAD3* [59]. ELF5 was also found to physically interact with AR in prostate cancer cells and repress its transcriptional activity. Furthermore, *ELF5* expression is induced upon AR activation in prostate cancer cells and is a transcriptional target of AR, suggesting a role in negative feedback regulation of AR signaling [60].

Similarly, ELF5 mRNA and protein expression is decreased in urothelial (bladder) cancers compared to the normal urothelium, and is inversely correlated with bladder cancer grade [61]. Downregulation of ELF5 in epithelial-like bladder cancer cell lines induced EMT, while its re-expression in mesenchymal-like T24 cells promoted MET [61]. The repression of ELF5 in urothelial cancers is linked to promoter methylation, and can be reversed by treatment with the DNA methyltransferase (DNMT) inhibitor 5-Azacytidine [61].

Finally, *ELF5* mRNA expression in ovarian cancer [62] and ELF5 mRNA and protein in renal cell carcinoma [63] is reduced compared to corresponding normal tissue, and re-expression of ELF5 in cell line models of these cancers inhibits cell proliferation and survival [62,63]. 

### 1.5. Role of EHF in Normal Tissue Homeostasis 

**Figure 7:**
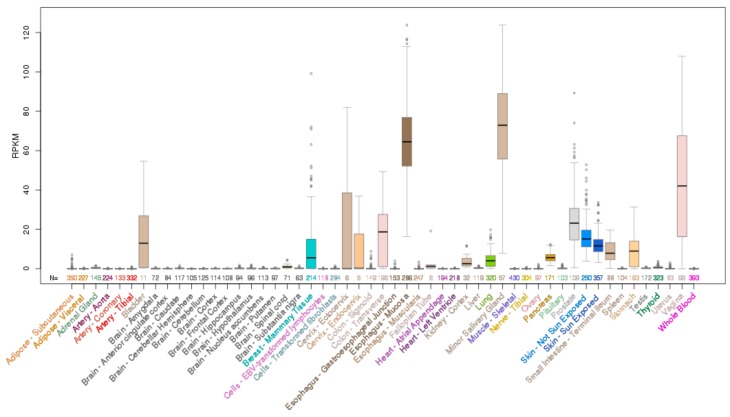
Expression of *EHF* mRNA across normal human tissues. Data obtained from UCSC genome browser.

*EHF* is located on chromosome 11p13 within close proximity to *ELF5* and encodes a 300 aa protein. Analysis of the mRNA expression pattern of *EHF* across normal human tissues indicates highest expression in the salivary gland, esophagus, vagina, prostate, colon, skin, bladder and breast (Figure 7). In contrast to the other ESE factors, the impact of *Ehf* deletion in mice has not been reported. 

#### 1.5.1. EHF in the Airway Epithelium

*EHF* has been studied extensively as a potential modifier gene of the severity of the cystic fibrosis phenotype. These studies stem from genome-wide association studies which identified a single nucleotide polymorphism (SNP) associated with the severity of lung disease in cystic fibrosis patients (rs12793173), which is located at chr11p13 within or close to potential *EHF* cis-regulatory elements [64]. Several subsequent studies sought to determine the role of EHF in bronchial epithelial cells where EHF is basally expressed, and is induced in response to inflammatory mediators [65]. Direct EHF targets in primary human bronchial epithelial cells are enriched for genes involved in lung pathology including EMT and wound response, and knockdown of EHF reduced wound closure in bronchial epithelial cells derived from both healthy and cystic fibrosis patients [66]. Most recently, a direct role for EHF in repressing *CFTR* expression was demonstrated in bronchial epithelial cells, and EHF binding to a regulatory region 35 kb upstream of the *CFTR* promoter was demonstrated by ChIP [67]. However, a clear model of how this SNP may contribute to the cystic fibrosis phenotype is yet to emerge. 

#### 1.5.2. EHF in the Skin

EHF plays an essential role in keratinocyte differentiation. Genome-wide profiling of enhancer regions in keratinocytes identified the *GGAA* Ets motif as the most enriched transcription factor binding site. As EHF was the ESE factor found to have the highest lineage-specific expression in stratified epithelia, its role in keratinocyte differentiation was directly investigated by knockdown in organotypic human epidermal tissue, revealing that EHF regulates ~400 genes in this tissue including several associated with keratinocyte differentiation [68]. 

#### 1.5.3. EHF in the Intestinal Epithelium

EHF mRNA and protein is highly expressed in the normal colon with maximal expression in the proliferative stem-cell compartment of the crypt base [69], and is among the ~500 genes maximally expressed in LGR5+ intestinal stem cells [70]. *Ehf* mRNA is also highly expressed in specialised M (microfold cells) cells which are located in the follicle-associated epithelium (FAE) which overlies Payer’s patches in the intestine. Here, M cells contribute to immune surveillance by mediating transcytosis of potential antigens from the intestinal lumen into the underlying immune cells [71]. *EHF* promoter activity was induced in the Caco-2 cell line model of FAE, and ectopic expression of EHF in this model induced expression of transcytosis associated genes. Notably however, EHF overexpression was not sufficient to induce M cell differentiation in this model, suggesting EHF may promote functional maturation of the FAE rather than M cell differentiation [72]. 

#### 1.5.4. EHF in Non-Epithelial Cells

While EHF mRNA and protein expression is highest in epithelial cells, there is also evidence that EHF is expressed in different components of the innate immune system, particularly dendritic cells (DCs) [73,74], where EHF is upregulated during cytokine-induced maturation of DCs, and knockdown of EHF impairs the maturation process [73]. 

EHF also plays a role in suppressing activation of mast cells, the major effector of IgE-driven hypersensitivity. TGFβ suppresses mast cell activation, inducing EHF mRNA and protein expression during this process. Overexpression of EHF in mast cells mimicked several of the TGFβ-induced transcriptional changes and inhibited mast cell degranulation, suggesting a direct role in TGFβ-mediated suppression of mast cell activity [75], although knockdown or knockout studies are still needed to confirm these findings. 

### 1.6. Role of EHF in Cancer 

**Figure 8:**
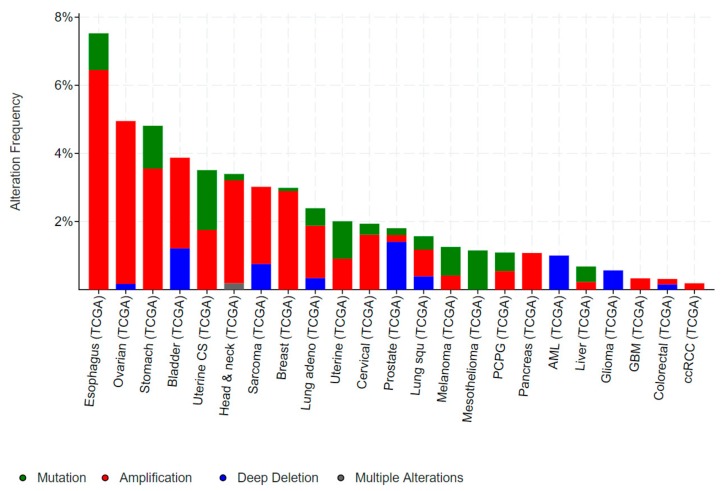
Genomic alterations in *EHF* in human cancers. Data from cancer bioportal. Only showing cancers in which >50 cases were analysed.

Mutations in *EHF* are relatively rare in human cancers and those which do arise are predominantly non-recurring missense mutations (Figure 8). However, occasional truncating mutations occur in gastric, uterine and cervical cancers, suggestive of a potential tumour suppressive role (Figure 8). Conversely, *EHF* amplifications occur in a subset of oesophageal, ovarian, stomach, bladder, and head and neck cancers, however, as discussed above, *EHF* is located within close proximity to *ELF5* and has an amplification and deletion profile which is very similar to *ELF5* (Figure 6), making it difficult to determine whether one, or perhaps both, transcription factors provides a selective advantage for these tumours.

#### 1.6.1. EHF in Gastric and Colorectal Cancer. 

The rate of *EHF* amplification in GC identified in large genomic studies is ~4%, however, Shi et al. reported *EHF* amplification in ~40% of cases, which was associated with worse outcome [76]. Nevertheless, consistent with an oncogenic role in this disease, EHF knockdown inhibited proliferation, survival, migration and invasion of GC cell lines in vitro and in vivo. In terms of mechanism, EHF was shown to drive GC progression through direct transactivation of the *HER2* promoter, and activation of downstream MAPK and PI3K/AKT signaling [76]. 

In CRCs, a study by Taniue et al. suggested that EHF is required for the survival of *TP53* wild-type CRC cell lines. Knockdown of EHF in the *TP53* wild-type HCT116 CRC cell line reduced expression of RUVBL1, an ATPase associated with chromatin remodeling and a repressor of TP53 expression [77]. However direct induction of TP53 following EHF knockdown was not demonstrated. 

#### 1.6.2. EHF in Thyroid Cancer

EHF mRNA and protein is expressed in papillary thyroid cancers (PTCs) and EHF knockdown in PTC cell lines inhibits cell proliferation, invasion and migration in vitro and in vivo. As observed in gastric cancers, *EHF* mRNA expression correlated with *HER2* and *HER3* expression in primary PTCs, and EHF was shown to directly bind and transactivate the *HER2* and *HER3* promoters in PTC cell lines [78]. 

#### 1.6.3. EHF in Ovarian Cancer

A tumour promoting role for EHF has also been suggested in ovarian cancer (OC), where *EHF* mRNA is overexpressed and correlates with poor overall survival [79]. Knockdown of EHF in OC cell lines inhibited cell proliferation, which was associated with reduced expression of Cyclin B1 and Cyclin D1, and upregulation of the cyclin-dependent kinase inhibitor, p21. EHF knockdown also inhibited migration and invasion of OC cell lines, and reduced expression of MMP9 [79]. 

#### 1.6.4. EHF in Prostate Cancer

In contrast to the tumour promoting roles described above, a tumour suppressive role for EHF is described in prostate, pancreatic and oesophageal cancers. In prostate cancer, EHF mRNA and protein expression is decreased, which is linked to methylation of a conserved CpG residue within the *EHF* promoter [80]. Loss of EHF expression is more pronounced in tumours with higher expression of cancer stem cell markers, and is associated with poorer patient outcome. Re-expression of EHF in prostate cancer cells inhibited clonogenic survival and induced apoptosis by directly driving caspase-3 expression [80]. Subsequent studies demonstrated that EHF re-expression in prostate cancer cells inhibited stem like properties and promoted epithelial differentiation by repressing EMT drivers, such as TWIST1, ZEB2, NANOG and POU5F1 [81]. This effect was mechanistically linked to EHF-mediated repression of the Lin28a and Lin28b RNA binding proteins, and re-expression of members of the *let-7* family of miRNAs [82]. EHF also represses IL-6 in prostate epithelial cells by directly binding the *IL-6* promoter, and EHF loss drives tumour progression by de-repression of IL-6, and subsequent stimulation of STAT3 signaling [82]. Finally, Kunderfranco et al. demonstrated that EHF contributes to tumour suppression of prostate cancer cells by repressing expression of *EZH2* and promoting expression of the tumour suppressor *Nkx3.1* [83].

#### 1.6.5. EHF in Pancreatic and Oesophageal Cancer

EHF protein expression is downregulated in pancreatic ductal adenocarcinomas (PDAC), which is associated with poorer differentiation grade and worse patient outcome. EHF knockdown in PDAC cell lines promoted cell motility and invasiveness, and increased metastasis in an orthotopic model, which was linked to repression of its direct transcriptional target, E-Cadherin [84].

In oesophageal squamous cell carcinoma (ESCC), the primary mechanism of EHF protein dysregulation is altered subcellular localization, where in contrast to its predominantly nuclear expression in the normal oesophageal epithelium, EHF is localized to the cytoplasm. While the mechanisms driving the cytoplasmic localization of EHF are yet to be defined, re-expression of EHF in ESCC cell lines which restored its nuclear expression, inhibited cell proliferation, colony formation, migration, and invasion [85].

### 1.7. Role of SPDEF in Normal Tissue Homeostasis 

**Figure 9:**
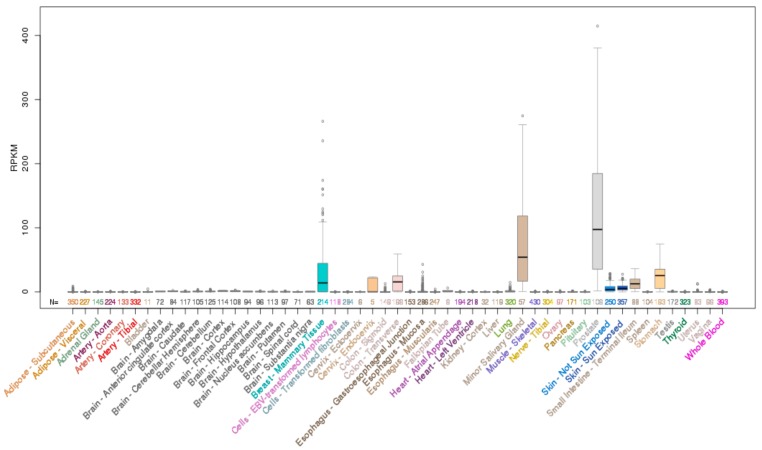
Expression of *SPDEF* mRNA across normal human tissues. Data obtained from UCSC genome browser.

Finally, *SPDEF* is localized to chromosome 6p21.31 and encodes a 335 aa protein. In contrast to ELF3, ELF5 and EHF, which preferentially bind to the *GGAA* motif, SPDEF preferentially binds to *GGAT* [86]. Analysis of *SPDEF* mRNA expression across normal human tissues indicates high expression in the prostate, salivary gland, stomach, colon and breast (Figure 9).

#### 1.7.1. *Spdef* Knockout Mice

Two mouse models of *Spdef* deletion have been generated [87,88]. The Clevers lab generated a *Spdef* knockout strain in which Exon 6 encoding the DNA binding domain was deleted. These mice were born at the expected Mendelian ratios and were fertile, however displayed reduced numbers and impaired terminal differentiation of goblet cells in the intestinal [87], conjunctival [89,90] and tracheobronchial mucosa [91]. Transgenic overexpression of SPDEF in nonciliated respiratory epithelial cells [92], or intestinal epithelial cells, induced expansion of goblet cells in these tissues supporting a role for SPDEF as a goblet cell lineage determinant [93].

Horst et al. developed an independent *Spdef^−/−^* strain in which exons 2-5 encoding the transactivation domain, the PNT domain, and most of the ETS DNA-binding domain were deleted [88]. These mice were also viable and fertile, although a modest decrease in the percentage of *Spdef^−/−^* mice was noted from heterozygote matings (18% vs. 25%). Notably, 40% of *Spdef^−/−^* mice developed thickening of the antral stomach by 4 months, and half of the *Spdef^−/−^* mice developed mucosal hyperplasia of the gastric antrum during the course of their lifetime, which was preceded by submucosal infiltration of inflammatory cells. Deletion of *Spdef* impaired differentiation of antral mucous gland cells, consistent with its common role in regulating differentiation of the goblet cell lineage in multiple tissues [88].

#### 1.7.2. SPDEF in the Prostate Epithelium 

While no phenotype in the normal prostate of *Spdef^−/−^* mice has been described, *SPDEF* mRNA is highly expressed in the terminally differentiated secretory luminal cells of the prostate epithelium [94]. SPDEF also interacts with the DNA binding domain of AR, and synergistically activates the *PSA* gene promoter [94]. 

#### 1.7.3. Role of SPDEF in Cancer 

**Figure 10:**
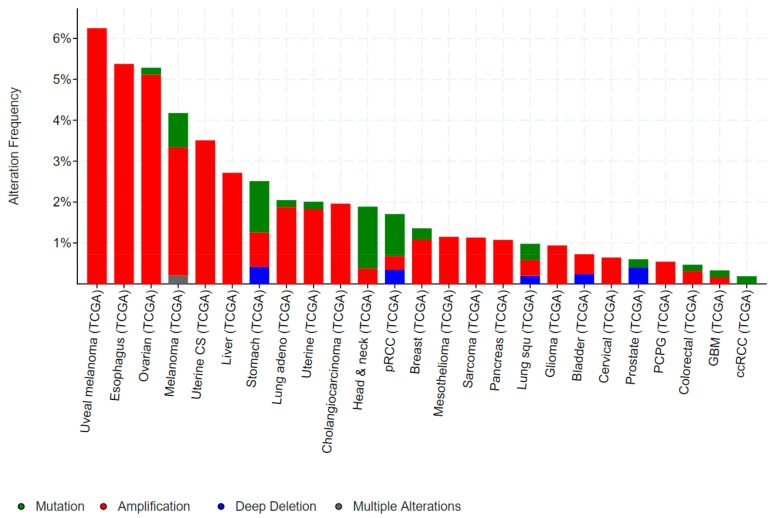
Genomic alterations in *SPDEF* in human cancers. Data from cancer bioportal. Only showing cancers in which >50 cases were analysed.

Mutations in *SPDEF* are rare in human cancers, with the majority of mutations being non-recurring missense mutations (Figure 10). One exception is gastric cancer, where truncating mutations in *SPDEF* are observed in a small subset of cases. The phenotype of *Spdef^−/−^* mice, which develop gastric mucosal hyperplasia [88], suggests these truncating mutations may be pathogenic. In addition, amplifications of *SPDEF* are found in a small subset of melanomas, esophageal, ovarian, uterine, lung and liver cancers (Figure 10), however the significance of these events in disease initiation or progression has yet to be directly tested. 

The functional role of SPDEF in progression of several cancers has been investigated, including CRC, HCC, bladder, prostate and ovarian cancer. In each of these cases, the majority of evidence suggests a tumour suppressive role, which are described below. 

#### 1.7.4. SPDEF in Colorectal Cancer

SPDEF mRNA and protein expression is decreased in primary CRCs and CRC cell lines compared to the normal colonic epithelium [95,96], and SPDEF re-expression in CRC cell lines induces cell cycle arrest, apoptosis and inhibits cell migration. Conversely, SPDEF knockdown in HT29 CRC cells increased cell proliferation [96]. These findings were validated in animal models, where adenoma formation was significantly increased when *Spdef^−/−^* mice were crossed to *Apc^Min/+^* mice or challenged with DSS/AOM [95]. Conversely, transgenic expression of *Spdef* in *Apc^Min/+^* mice reduced the mitotic rate of these adenomas [95].

Mechanistically, overexpression of SPDEF in mouse intestinal adenomas or CRC cell lines decreased WNT signaling [95]. A physical interaction between the PNT domain of SPDEF and the Armadillo repeats on β-catenin was subsequently demonstrated, which disrupted β-catenin binding to TCF/LEF proteins and displaced β-catenin from promoter/enhancer regions of cell cycle genes, inducing cellular quiescence [97]. 

#### 1.7.5. SPDEF in Hepatocellular Cancer 

SPDEF mRNA and protein expression is reduced in HCC, particularly in poorly differentiated tumours, and is associated with worse patient outcome [98]. Knockdown of SPDEF in HCC cell lines increased cell proliferation, survival and invasion in vitro and tumour growth and metastasis in vivo. The increased invasion and metastasis are likely due to increased expression of the EMT driver, SLUG. Comparatively, overexpression of SPDEF in HCC cell lines decreased proliferation and increased apoptosis [98]. 

#### 1.7.6. SPDEF in Bladder Cancer

SPDEF mRNA and protein expression is decreased in bladder cancer, particularly in high grade tumours, and re-expression of SPDEF in bladder cancer cell lines inhibited proliferation and invasion in vitro, and reduced xenograft growth in vivo. SPDEF re-expression also reduced EMT markers, while SPDEF knockdown induced the opposite effect [99].

#### 1.7.7. SPDEF in Prostate Cancer

In prostate cancer, loss of SPDEF protein correlates with poor differentiation and worse patient outcome, which is also reflected in prostate cancer cell lines where more aggressive lines express lower SPDEF [100,101]. Re-expression of SPDEF in SPDEF-negative PC3 cells inhibited cell migration, invasion and metastasis [100,102], while SPDEF knockdown in LNCaP cells promoted these processes [100,102]. Finally, deletion of *Spdef* in mice increased prostate tumour formation in the TRAMP model, while transgenic overexpression of *Spdef* reduced tumorigenesis [103]. Mechanistically, *Spdef* directly repressed expression of Foxm1, a key transcription factor required for tumour cell proliferation, and reduced expression of multiple Foxm1 target genes involved in cell cycle progression including *Cdc25b*, Cyclin B1, Cyclin A2 [103]. 

*SPDEF* is also regulated by AR signalling in prostate cancer, which has both beneficial and potentially detrimental effects when AR signaling is inhibited by androgen deprivation therapy (ADT). Specifically, AR-mediated activation of *SPDEF* repressed expression of *TGFBI* and *CCL2*, key drivers of prostate cancer metastasis [104,105]. ADT relieves this repression and inadvertently promotes metastasis by increasing *TGFBI* and *CCL2* expression, providing an example of how a therapy which blocks growth of the primary tumour may paradoxically promote metastasis. 

#### 1.7.8. SPDEF in Ovarian Cancer

Initially, SPDEF mRNA and protein was reported to be overexpressed in ovarian cancer (OC), particularly serous epithelial ovarian tumours [106,107]. However, SPDEF mRNA and protein expression was later found to be lower in OC compared to non-neoplastic tissue, and retention of SPDEF found to be a favourable prognostic marker [108]. Supporting a tumour suppressive role, SPDEF re-expression in SPDEF-negative OC cells inhibited cell proliferation and induced apoptosis, which was associated with reduced expression and promoter activity of the pro-survival gene, survivin (*BIRC5*) [108]. 

#### 1.7.9. SPDEF in Breast Cancer

Similar to other ESE members, the role of SPDEF in breast cancer also varies according to the molecular subtype of the disease. In TNBC, a tumour suppressive role is suggested by the loss of SPDEF protein in TNBC cell lines [109,110], which has been linked to inhibition of translation of *SPDEF* mRNA by *miR-204* and *miR-510* [111]. Furthermore, SPDEF re-expression inhibits the growth and migration of the TNBC cell line, MDA-MB-231 [109,110]. 

Conversely, in ER+ breast cancers, *SPDEF* mRNA is overexpressed and correlates with poor overall survival [112,113]. Similarly, knockdown of SPDEF in multiple luminal cell lines inhibited proliferation, increased basal apoptosis, and increased sensitivity to the ER antagonists, tamoxifen and fulvestrant [114]. This finding was subsequently validated in a genome-wide shRNA screen of multiple breast cancer cell lines which identified *SPDEF* and *FOXA1* as the two most essential genes required for the growth and survival of luminal/HER2 cell lines [115]. Similar to the findings reported for ELF5, SPDEF expression was also elevated in MCF7 cells made resistant to tamoxifen, and SPDEF knockdown in these lines resulted in extensive apoptosis, suggesting SPDEF may also play a role in resistance to endocrine therapy [114]. 

### 1.8. ESE Transcription Factors as Therapeutic Targets in Cancer

As outlined in this review, there is now strong evidence that the ESE transcription factors contribute to the progression of several cancers, functioning as either tumour promoters or inhibitors, depending on the tumour type, and in some cases tumour subtype. While inactivating mutations in these factors occurs occasionally in cancers, in the majority of cases ESE factors impact tumour progression as a consequence of their altered expression. In cases where tumour progression is driven by their overexpression, developing treatments to inhibit ESE function represents a logical approach. In this context, a potential advantage of targeting ESE transcription factors for cancer treatment is their relatively restricted expression across tissues, which may limit toxicities. Proof of concept for this approach lies in the effective therapeutic targeting of ER and AR in breast and prostate cancer, which exploits the lineage-specific functionality of these transcription factors rather than their genetic alteration, to create a therapeutic window. The potential for targeting specific ESE factors is also supported by the survival of *Elf3^−/−^* and *Spdef^−/−^* mice. 

Comparatively, where loss of expression of ESE transcription factors drives tumorigenesis, therapeutic opportunities may be limited to re-inducing their expression or targeting the pathways or cellular processes which are activated as a consequence. In this context, a recurring theme is the requirement of several ESE factors for maintenance of the epithelial state of many cancers. Therapies which re-induce ESE factors may therefore have specific use in metastasis prevention, or may provide secondary benefit subsequent to re-epithelialization of cancers, such as improving drug response. Potential strategies by which ESE factors may be inhibited or activated are discussed below. 

#### 1.8.1. Direct Targeting

While the direct targeting of transcription factors has traditionally been considered challenging due to the difficulty in directly modulating protein/DNA binding, a number of clinical successes have highlighted the therapeutic value of transcription factor targeting for cancer treatment. This includes the use of inhibitors of the nuclear receptors ER, AR and GR (Glucocorticoid receptor) for the treatment of breast, prostate and lymphoid cancers, respectively, and the use of all *trans* retinoic acid for treating acute promyelocytic leukemia patients harboring *PML-RARα* gene fusions [116]. 

Although there are currently no small molecules that can directly target ESE transcription factors, several approaches can be envisioned. These include the use of decoy oligonucleotides which mimic ETS binding sites to saturate ESE factors as previously tested for Ets1 in a model of gastric cancer [117], or specific DNA-binding compounds which block transcription factor-DNA interactions as described for Sp1 [118], NF-κB [119], EVI1 [120] and the ETS factor, ERG1 [121]. However, a likely limitation of this approach is non-specific DNA targeting which may particularly impact the targeting of ESE factors where multiple ETS family members bind the same DNA motif. 

Several ESE factors co-operate with other transcription factors or co-factors to modulate gene expression which also creates therapeutic opportunities. For example, ELF3 interacts with AR and ER and inhibits their transcriptional activity [35,42]. Similarly, SPDEF and ELF3 bind to and inhibit the transcriptional activity of β-catenin in CRC cells [33,97]. Compounds that promote or mimic these interactions may therefore have therapeutic potential. Notably, Gajulapalli et al. performed molecular docking studies to map the minimal region of ELF3 which interacts with the DNA binding domain of ERα. Based on these findings, they developed a synthetic 17 aa ELF3 peptide of this region which inhibited the DNA binding activity of ERα and proliferation of ERα positive but not ERα negative breast cancer cell lines [35]. 

#### 1.8.2. Indirect Targeting

An alternative strategy for targeting transcription factors is to disrupt their interaction with key transcriptional co-factors. These include epigenetic regulators such as histone aceyltransferases, deacetylases, methylases and demethylases. These regulators are recruited by transcription factors to modify surrounding chromatin, or to induce post-translational modification of the transcription factors themselves. For example, ELF3 interacts with the histone acetyltransferases p300 and CBP, which increases its transcriptional activity [122,123]. Further studies to identify the transcriptional activators and repressors which interact with ESE factors represents a promising field of investigation, as a number of small molecule inhibitors of transcriptional co-repressors and co-activators have now been generated, with several in clinical use [124]. 

#### 1.8.3. Targeting Pathways Altered by ESE Factors in Cancer

In cases where ESE factors are genetically inactivated, targeting the pathways and processes which are consequently activated may represent a viable strategy. For example, loss of EHF in prostate cancer activates JAK/STAT signaling and sensitizes cells to treatment with a JAK2 inhibitor [82]. Conversely, overexpression of EHF drives HER2 expression in gastric and thyroid cancers, sensitizing these tumours to HER2 inhibition, while in lung cancer cell lines, ELF3 overexpression activates the PI3K/AKT and MAPK pathways and sensitized lung cancer cells to inhibitors of these pathways [43]. 

#### 1.8.4. Re-inducing Expression of ESE Factors

A further mechanism of ESE transcription factor suppression in cancer is via promoter methylation, which has been reported for *EHF* in prostate [80] and pancreatic cancer [84], and for *ELF5* in bladder cancer [61]. In these cases, treatment with a demethylating agent re-induced their expression [61,84]. A limitation of this approach, however, is the broad ranging transcriptional effects of demethylating agents. Importantly, promoter methylation can be a reflection of altered transcription [125], therefore detailed study of the specific mechanisms which predispose these promoters to transcriptional downregulation and methylation may identify more specific strategies for re-inducing their expression. 

## 2. Conclusions

As outlined in this review, the ESE transcription factors play a critical role in the development and differentiation of a number of epithelial tissues. There is also now strong evidence directly implicating either the gain of loss or these transcription factors in cancer progression, which is dependent on tumour type and in some cases, tumour subtype. While additional studies, particularly confirmation of findings in mouse models of cancer, are still required in some cases, the challenge now is to develop novel cancer treatments focused on either direct targeting of these factors, re-inducing their expression, or targeting the pathways disrupted as a consequence of ESE factor deregulation.
